# Supplementary material for: The Safety Action Feedback and Engagement (SAFE) Loop: Initial Testing and Refinement of a Novel Intervention to Enhance Hospital Incident Reporting and Patient Safety
Source: medRxiv. 2025 Jun 6:2025.06.03.25328744. Preprint. [Version 1] doi: 10.1101/2025.06.03.25328744 (PMC12154985; doi:10.1101/2025.06.03.25328744)
Supplement: Supplement 1 [file media-1.pdf]

Appendix:

Measures Derived from Survey

	**Type**	**Scale**	**Timing**
Confidence in Reporting	Composite measure, novel	Percent positive response (0-100%)	All
Communication About Error	Composite measure, AHRQ SOPS	Percent positive response (0-100%)	All
Reporting Patient Safety Events	Composite measure, AHRQ SOPS	Percent positive response (0-100%)	All
Blame-free Culture	Individual measure, AHRQ SOPS	Percent positive response (0-100%)	All
Incident Report Submissions	Individual measure, AHRQ SOPS	0-4+, Median, IQR	All
Time per Report	Individual measure, novel	Continuous (0-90)	All

Survey Instrument

**Item Stem**	**Response Options**	**Source**
How often do the following things happen in your unit?		
1. Nurses are informed about errors that happen in this unit.	Response Options:<br>1 – Never<br>2 – Rarely<br>3 – Sometimes<br>4 – Most of the time<br>5 – Always<br>6 -- Does not apply or don't know	AHRQ SOPS Hospital Survey 2.0, topic 6, communication about error, Q C1.
2. When errors happen in this unit, nurses discuss ways to prevent them from happening again.		AHRQ SOPS Hospital Survey 2.0, topic 6, communication about error, Q C2.
3. In this unit, nurses are informed about changes that are made based on incident reports.		AHRQ SOPS Hospital Survey 2.0, topic 6, communication about error, Q C3.
4. When staff make errors, this unit focuses on learning rather than blaming individuals.		AHRQ SOPS Hospital Survey 2.0, topic 4, response to error, Q A10.
5. When a mistake is caught and corrected before reaching the patient, how often is this reported using the incident reporting system?		AHRQ SOPS Hospital Survey 2.0, topic 8, reporting patient safety events, Q D1.
6. When a mistake reaches the patient and could have harmed the patient, but did not, how often is this reported using the incident reporting system?		AHRQ SOPS Hospital Survey 2.0, topic 8, reporting patient safety events, Q D2.
How much do you agree or disagree with the following statement about your unit?		
7. How confident are you in knowing when to complete an incident report for a medication safety event?	Consider the following questions related to completing an incident report for a medication safety event.<br>Response Options:<br>1 – Not at all confident<br>2 – Slightly confident<br>3 – Moderately confident<br>4 – Very confident<br>5 – Extremely confident	Novel
8. How confident are you in knowing what information to include when completing an incident report for a medication safety event?		Novel

9. In the last 6 months, how many medication safety events have you personally reported via the incident reporting system?	1 – None<br>2 – 1<br>3 – 2<br>4 – 3<br>5 – 4 or more	AHRQ SOPS Hospital Survey 2.0, topic [11], number of events reported, Q D3.
[If response to item above was 2 through 5]<br>10. Please think of the last time you completed an incident report for a medication safety event. How many minutes did it take you to complete the report?	[Response options in increments of 5 min, e.g., 5, 10, 15, up to 90…)	Novel

Coding of Measures for Analysis:

Composite measure "Confidence in Reporting:" Use methods below for calculating composite measures. Positive responses: very confident or extremely confident.

- How confident are you in knowing when to complete an incident report for a medication safety event?
- How confident are you in knowing what information to include when completing an incident report for a medication safety event?

Composite measure "Communication About Error:" The extent to which staff are informed when errors occur, discuss ways to prevent errors, and are informed when changes are made. Positive responses: most of the time or always. (Q C1-C3)

- Nurses are informed about errors that happen in this unit.
- When errors happen in this unit, nurses discuss ways to prevent them from happening again.
- In this unit, nurses are informed about changes that are made based on incident reports.

Composite measure "Reporting Patient Safety Events:" The extent to which mistakes of the following types are reported: (1) mistakes caught and corrected before reaching the patient and (2) mistakes that could have harmed the patient but did not. (Q D1-2)

- When a mistake is caught and corrected before reaching the patient, how often is this reported using the incident reporting system?
- When a mistake reaches the patient and could have harmed the patient, but did not, how often is this reported using the incident reporting system?

Individual measure "Blame-free Culture:" When staff make errors, this unit focuses on learning rather than blaming individuals. Positive responses: most of the time or always.

Individual measure "Incident Report Submissions:" In the last 6 months, how many medication safety events have you personally reported via the incident reporting system?

Individual measure "Time per Report:" Please think of the last time you completed an incident report for a medication safety event. How many minutes did it take you to complete the report?

Methods for calculating percent positive response and, if applicable, creating a composite measure:

- Identify respondents to the survey overall. Exclude non-respondents.
- For an individual item of interest,
  - Identify and count responses. Exclude those with missing responses and does not apply/do not know responses to that item.
  - Identify and count positive responses.

    - Calculate the percent positive response as the count of positive responses divided by the total count of responses.
- To calculate the composite measure, average the percent positive scores for each item included in the composite measure.
